# Assessing bias in the causal role of HPV in oral cancer: A systematic review and meta‐analysis

**DOI:** 10.1111/odi.15062

**Published:** 2024-07-02

**Authors:** Gagandeep Kaur, Tami Yap, Rishi Ramani, Michael McCullough, Ankur Singh

**Affiliations:** ^1^ Centre for Epidemiology and Biostatistics, Melbourne School of Population and Global Health University of Melbourne Melbourne Victoria Australia; ^2^ Melbourne Dental School University of Melbourne Melbourne Victoria Australia

**Keywords:** HPV, human papillomavirus, oral cancer

## Abstract

**Objective:**

High‐risk human papillomaviruses (HPV) are an established cause of oropharyngeal cancer. Their relationship with oral cancer remains unclear with detection ranging from 0% to 100%. HPV DNA detection or evidence of exposure alone is insufficient to conclude causality. This systematic review assesses the extent of bias in studies of HPV detection in cancers of the oral cavity.

**Methods:**

PubMed, Ovid MEDLINE, EMBASE, and PsycInfo databases were searched for observational studies reporting the effect of HPV in oral cavity specific cancers.

**Results:**

All 15 included studies presented HPV DNA detection or serum HPV‐antibodies, none included mRNA E6/E7 analysis. Cases with oral cancer had 5.36 times (95% CI 3.29–8.72) higher odds of having HPV detected compared to controls. The odds of HPV detection were higher in cell‐based (OR 6.93; 95% CI 0.82–58.55) and tissue samples (OR 5.28; 95% CI 3.41–8.18) than blood‐based samples (OR 3.36; 95% CI 1.53–7.40).

**Conclusion:**

When cancer site is clearly differentiated between oropharynx and oral cavity, 12 studies showed strong association between HPV and oral cancer, but the available estimates lack internal validity due to inconsistent measurements, high confounding, and lack of gold standard testing. There is not high‐quality evidence to conclude a causal relationship of HPV with oral cancer.

## 1 BACKGROUND

Oral cancer (OC) or oral cavity cancer includes cancers that develop in the tissues of the oral cavity, which includes the tongue, upper and lower gingiva, oral floor, palate, and buccal mucosa. Oral potentially malignant disorders (OPMDs) are precursor mucosal lesions with the potential for malignant transformation (Irani, 2016). Human papillomavirus (HPV), a common sexually transmissible infection, has two categories: low‐risk HPV, usually responsible for benign lesions like warts and high‐risk HPV (such as HPV‐16 and HPV‐18), which is associated with OPMDs, oral, oropharyngeal and cervical cancers (Herrero et al., 2003; Syrjanen et al., 2011).

Literature suggests a strong causal relationship between HPV and oropharyngeal cancer (OPC), but the role of HPV in OC is less clear (Herrero et al., 2003; Hobbs et al., 2006). The prevalence of HPV in oral cavity tumours has been reported as ranging from 0% to 100% (Dalakoti et al., 2019). Some reviews on HPV and OC conclude that a strong causal relationship exists between HPV and oral squamous cell carcinoma (OSCC) which is the most common type of OC, while others are inconclusive (Appendix S1).

Information bias or misclassification is defined as an error in collecting or measuring data on exposure, outcome or confounding factors (Althubaiti, 2016). It can lead to over‐estimation or under‐estimation of the effect of HPV on OC due to which a causal relationship cannot be established with certainty. Consequently, this can create ambiguity on effectiveness of interventions like HPV vaccine targeting the virus for prevention of OC or OPMDs. Key sources of information bias in studies of HPV and OC include:

1. Choice of sampling and detection methods (Appendix S2) leading to exposure misclassification.
*Sampling methods*: Choice of sampling of tissue can impact the accuracy of HPV measurement. For instance, a tumour tissue sample provides a site‐specific measurement of HPV as compared to a serum sample that can represent infection or history of infection elsewhere in the body.
*Detection methods*: P16 evaluation is the most commonly used technique due to its cost effectiveness and ease of use (Jakobsen et al., 2021; Lewis Jr. et al., 2018). However, the use of p16 immunochemistry may not provide accurate results (Gotz et al., 2016). Recent evidence indicates that L1/E6/E7 antibody seropositivity may be more effective than p16 IHC, but antibodies may be elicited by past infection or vaccination (Blatt et al., 2021). DNA analysis by in situ hybridisation or by polymerase chain reaction (PCR) detects the presence of viral DNA, but it cannot determine whether the virus is oncogenically active. Presence of E6/E7 mRNA provides evidence for transcriptional activation of the viral oncoproteins E6 and E7 that is necessary for malignant transformation of HPV positive cells (Yim & Park, 2005). Evidence shows that presence of transcriptionally active HPV is rare in OC and p16 IHC may not be an appropriate marker of HPV presence and activation (Belobrov et al., 2018). Therefore, E6/E7 HPV mRNA evaluation is the widely accepted gold standard technique for HPV detection (Jakobsen et al., 2021; Lewis Jr. et al., 2018).
  Measurement of exposure may be subject to differential or non‐differential misclassification depending on study design. Differential misclassification of HPV status is not possible in cohort study designs using laboratory‐based testing but can occur in case–control studies that employ different methods of sampling and analysis in cases and controls. This can bias the effect estimate toward or away from the null, leading to over‐estimation or under‐estimation of the role of HPV in OC. Non‐differential misclassification occurs when HPV status is similarly misclassified among cases and controls and may depend on the sensitivity and specificity of the diagnostic method. It can bias the effect estimate towards null.
2. Poorly defined anatomical subsites leading to outcome misclassification. It is important to have clearly defined outcome subsites. Mixing of pharyngeal sites like base of tongue in oral cancer studies can lead to overestimation of the rate of HPV in OC as HPV has higher prevalence in OPCs (Herrero et al., 2003).

Recent research does not support the involvement of HPV in OC in individuals unexposed to traditional risk factors like tobacco or alcohol (Fiedler et al., 2023). Given the wide variability in reported HPV prevalence in oral cavity tumours and the inconclusive findings of existing reviews, ambiguity about HPV's causal role in cancers specific to the oral cavity persists. Assessing key sources of bias (including information bias, selection bias, and confounding) in studies of HPV and OC is essential. Information bias is particularly important as misclassification of both HPV status and cancer site is possible. No existing reviews have assessed the validity of existing causal conclusions by focussing specifically on misclassification in studies reporting the association between HPV and OC.

This systematic review fills this critical gap by reviewing studies that clearly differentiate between oropharyngeal and oral cavity cancer sites and by addressing potential misclassification of HPV exposure. The aim of this systematic review was to summarise the evidence on the effect of HPV on OC and to investigate the presence of information bias in observational studies investigating HPV detection in cancers specific to the site of oral cavity.

## 2 METHODS

This systematic review followed a protocol published in the Prospective Register for Systematic Reviews (PROSPERO) (CRD42022327522) and is reported according to the guidelines in the Preferred Reporting Items for Systematic Reviews and Meta‐analyses (PRISMA) statement (Page et al., 2021) (Appendix S3).

### 2.1 Eligibility criteria

Observational studies assessing the effect of HPV on cancers of the oral cavity and OPMD's were included. Only studies that clearly stated the outcome site as located in the oral cavity were included. This includes cancer in the following oral sites: the anterior two thirds of the tongue, gingivae, oral floor, hard palate, buccal mucosa, and retromolar trigone. Studies reporting measures of association were included while studies that only reported measures of frequency were excluded. This is important as measures of frequency only tell us how widespread a disease is in a population, while measures of association help us understand the strength and direction of relationships between exposures and outcomes.

Studies that did not have a comparator group or lacked healthy individuals as a control group were excluded. Studies where participants had reported co‐infection by another oncogenic virus, such as Epstein–Barr virus, were excluded. Studies with cancer of the pharynx or oropharynx as sole outcome were excluded. Studies of head and neck cancer where oral site data were not reported separately, or studies where the total effect of HPV exposure on OC was unclear and could not be extracted, were excluded. Cross‐sectional studies, case reports, case series, and conference abstracts were excluded. No exclusions were made based on gender designation or geographical location.

### 2.2 Search strategy

The search strategy was designed to include terms relevant to the exposure (HPV), site (oral, mouth), and outcome (cancer, precancers, OPMDs). A comprehensive literature search was carried out using the following electronic databases: PubMed, Ovid MEDLINE, EMBASE, and PsycInfo. The search strategy was developed for PubMed and modified for use in other electronic databases (Appendix S4). The search strategy for other electronic databases is listed in Appendix S5.

### 2.3 Study selection and data extraction

Following the literature search, titles and abstracts of retrieved studies were screened by two reviewers (GK and RR) independently and relevant studies were selected for full text review based on inclusion and exclusion criteria. After full text review, data relating to general study characteristics, exposure, outcome, effect size, and confounding factors were extracted using a data extraction form (Appendix S6). This template was first piloted in 10% of included studies.

### 2.4 Risk of bias

Risk of bias was assessed using the Risk Of Bias In Non‐randomized Studies‐of Exposures (ROBINS‐E) tool that determines bias across seven domains including: confounding, selection of participants into the study, classification of exposures, departure from intended exposures, missing data, measurement of outcomes, and selection of the reported result. Overall bias for each study was judged as low, moderate, serious, or critical. Age, sex, tobacco, and alcohol consumption were included as the minimum set of confounders on the effect of HPV on OC, in the analyses based on a directed acyclic graph (Appendix S7).

### 2.5 Statistical analysis

A meta‐analysis of pooled estimates was performed using a random effects model. The results were reported as pooled estimates with 95% confidence intervals. Heterogeneity was quantified between studies with the *I*‐squared statistics. Subgroup analysis was conducted according to site specific measurement. Data were analysed using Stata (StataCorp. 2021. Stata Statistical Software: Release 17. College Station, TX: StataCorp LLC.).

## 3 RESULTS

### 3.1 Study selection

The study selection process is summarised in a PRISMA flowchart (Appendix S8). A total of 5584 studies were retrieved from the database search and imported into Covidence for screening. Title and abstract screening was undertaken for 2943 studies. Of these, 103 full text studies were selected and assessed for eligibility with 20 studies finally included in the review (Anaya‐Saavedra et al., 2008; Applebaum et al., 2007; Chang et al., 2003; Chen et al., 2002, 2006, 2016; Dalla Torre et al., 2015; Giovannelli et al., 2002; González‐Ramírez et al., 2013; Hansson et al., 2005; Kerishnan et al., 2016; Kingsley et al., 2021; Luo et al., 2007; Majumder et al., 2009; Mork et al., 2001; Pintos et al., 2008; Ribeiro et al., 2011; Saini et al., 2011; Szarka et al., 2009; Tsimplaki et al., 2017). The most common reason for exclusion was a lack of measure of association (*n* = 49). We further excluded studies with only crude estimates, and one study where the odds ratio could not be calculated due to the absence of positive cases. Subsequently, 16 estimates from 15 studies were included in the meta‐analysis (Refer Table 1).

**TABLE 1:** Study characteristics.

Author (year)	Country	Study design	Age (years)^a^	Outcome	Exposure	Adjusted OR (95%CI)	Minimum set of confounders				Sample type		Detection method
							Age	Sex	Tobacco	Alcohol	Cases	Controls	
Anaya‐Saavedra et al. (2008)	Mexico	Case–control	Range 27–86	OSCC	High‐risk HPV	5.77 (2.41–13.81)	No	No	Yes	Yes	Tissue	Surface cells	DNA analysis
Applebaum et al. (2007)	USA	Case–control	Mean 60.25	OSCC	HPV16	1.70 (1.02–2.84)	Yes	No	No	No	Blood	Blood	HPV – L1 antibodies
Chen et al. (2002)	Taiwan	Case–control	N/R	OSCC	HPV16	11.21 (1.22–103.08)	Yes	Yes	Yes	No	Tissue	Tissue	DNA analysis
Chen et al. (2016)	China	Case–control	Range 20 (±5)–80 (±5)	OSCC	HPV16/18	7.21 (2.61–19.90)	Yes	Yes	Yes	Yes	Tissue	Surface cells	DNA analysis
Dalla Torre et al. (2015)	Austria	Case–control	Mean 28.95 Range 19–50	OPMDs	High‐risk HPV	4.00 (1.81–8.84)	Yes	Yes	Yes	Yes	Surface cells	Surface cells	DNA analysis
Giovannelli et al. (2002)	Italy	Case–control	Mean 48 Range 19–50	OSCC, OPMDs	HPV	55.02 (9.17–330.12) 7.83 (2.22–27.62)	Yes	Yes	Yes	Yes	Surface cells	Surface cells	DNA analysis
González‐Ramírez et al. (2013)	Mexico	Case–control	N/R	OSCC	High‐risk HPV	5.80 (1.31–25.62)	No	No	Yes	Yes	Tissue	Surface cells	DNA analysis
Hansson et al. (2005)^b^	Sweden	Case–control	Range 33–89	OSCC	High‐risk HPV	27.24 (7.60–97.61)	No	No	Yes	Yes	Oral fluid	Oral fluid	DNA analysis
Kerishnan et al. (2016)	Malaysia	Case–control	Mean 48.9	OSCC	HPV16	13.59 (3.89–47.49)	Yes	Yes	No	No	Blood	Blood	HPV 16 specific IgG and IgM
Majumder et al. (2009)	India	Case–control	Mean 49.75	OSCC, OPMDs	HPV16/18	5.50 (1.60–18.95) 2.80 (1.20–6.52)	Yes	Yes	Yes	No	Tissue	Surface cells	DNA analysis
Mork et al. (2001)^b^	Norway, Finland & Sweden	Case–control	N/R	OSCC	HPV16	2.91 (1.33–6.37)	No	No	Yes	No	Blood	Blood	HPV antibodies
Pintos et al. (2008)^c^	Canada	Case–control	N/R	OSCC	HPV	1.29 (0.28–5.91)	Yes	Yes	Yes	Yes	Surface cells	Surface cells	DNA analysis
Pintos et al. (2008)^d^	Canada	Case–control	N/R	OSCC	HPV16	3.93 (0.88–17.58)	Yes	Yes	Yes	Yes	Blood	Blood	HPV antibodies
Ribeiro et al. (2011)	Central Europe & Latin America	Case–control	N/R	OSCC	HPV16	1.82 (0.09–36.95)	Yes	Yes	Yes	Yes	Blood	Blood	HPV antibodies
Saini et al. (2011)	Malaysia	Case–control	Mean 49.1	OSCC	HPV	3.57 (1.63–7.80)	Yes	Yes	Yes	Yes	Tissue	Surface cells	DNA analysis
Tsimplaki et al. (2017)	Greece	Case–control	Mean 54.7	OSCC	HPV	5.30 (1.00–27.99)	Yes	Yes	Yes	Yes	Surface cells	Surface cells	DNA analysis

Abbreviations: FOM, other oral cavity ORs; N/R, not reported.

^a^ Age is presented as mean or range (±range for controls where available).

^b^ Pooled oral tongue.

^c^ Surface cell‐based sample.

^d^ Blood‐based sample.

### 3.2 Study characteristics

Key characteristics of included studies are presented in Table 1. All 15 studies included in the meta‐analysis were case–control studies and reported odds ratios as effect estimates. Most of the studies were based in Europe and North America, with one partly based in South America and none from Africa. As shown in Table 1, in the 15 included studies, the reported outcomes included OC (*n* = 12), OPMDs (*n* = 1) or both (*n* = 2). There were four studies with an overall measure of HPV/any HPV as the exposure. For studies that reported high‐risk (HPV 16 and/or 18) and low‐risk HPV effect sizes separately (*n* = 4), high‐risk HPV estimates were selected for the meta‐analysis, assuming a high likelihood of cancer association. Only seven adjusted for the minimum set of confounders. The overall risk of bias using ROBINS‐E was judged to be serious in most of the included studies with only two studies having a moderate risk of bias (Appendix S9) (Chen et al., 2016; Pintos et al., 2008). The most common domains with a serious risk of bias were confounding (*n* = 13) and selection of participants in the study (*n* = 10). The detection methods used in the studies include DNA analysis using tissue biopsy or exfoliated cells and antibody detection using oral fluid or blood‐based samples (Table 1). None of the included studies used mRNA analysis or p16 IHC for detection.

### 3.3 HPV and oral cancer

**FIGURE 1:**
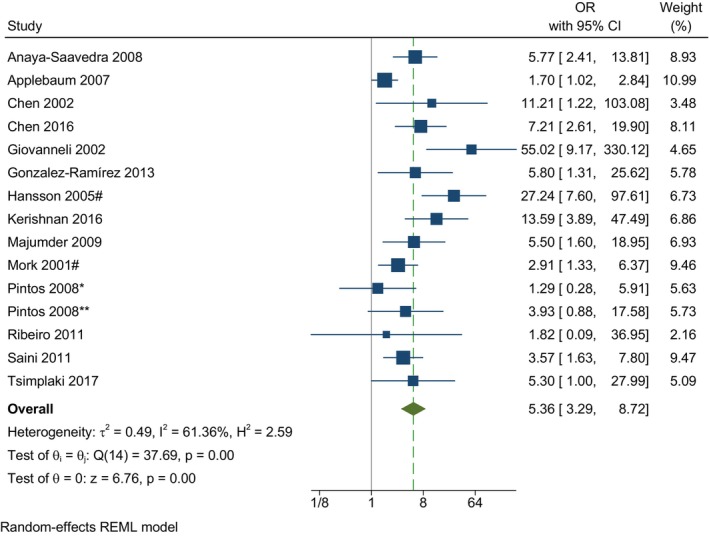
Forrest plot showing the pooled effect of HPV on oral cancer. Data are presented as odds ratio for each study (blue boxes), 95% CIs (horizontal lines) and summary as odds ratio with 95% CI (green diamond). *Surface cell‐based sample; **Blood‐based sample; # Pooled oral tongue, FOM, other oral cavity.

Of the 16 estimates from 15 included studies, 12 showed a strong association between HPV and OC while estimates from three studies (four estimates) were unclear (Table 1). Meta‐analysis for the effect of HPV on oral cancer showed that cases with OC had 5.36 times (95% CI 3.29–8.72) higher odds of having positive HPV detection when compared to controls (Figure 1). According to the overall *I*‐squared value of 61.36%, substantial heterogeneity was found between studies.

### 3.4 HPV and OPMDs

For OPMDs, cases had 3.92 times (95% CI 2.31–6.62) higher odds of positive HPV exposure as compared to the control group (Appendix S10).

### 3.5 Subgroup analysis

Out of the 16 estimates (from 15 studies) in the primary meta‐analysis, four provided an overall measure of HPV/ any HPV as the exposure, that is, did not specify if the HPV was 16, 18 or high‐risk only. A subgroup analysis for studies which only tested for HPV16, HPV 18 or high‐risk HPV only showed that cases with OC had 5.39 times (95% CI 3.14–9.24) higher odds of having positive HPV detection when compared to controls (Appendix S11). This result was not substantially different from the primary analysis (OR 5.36; 95% CI 3.29–8.72).

A subgroup analysis by detection method showed that the odds of positive HPV exposure in cases compared to controls was higher when using DNA analysis (OR 6.79; 95% CI 3.88–11.87) compared to HPV antibody serological testing (OR 3.36; 95% CI 1.53–7.40) (Appendix S12).

A subgroup analysis by sampling method used for case samples showed that the odds of HPV exposure in cases compared to controls was higher for studies using samples from exfoliated surface cells (OR 6.93; 95% CI 0.82–58.55) and tissue (OR 5.28; 95% CI 3.41–8.18) than studies with blood‐based samples (OR 3.36; 95% CI 1.53–7.40) (Appendix S13).

Upon investigating whether studies that undertook DNA analysis used similar or different types of samples for cases and controls, it was found that there was a wide variation in the effect estimates of studies that used the same method for cases and controls (*I* ^2^ = 69.10%) (Appendix S14). The odds of HPV in this subgroup (OR 10.15; 95% CI 2.68–38.39) was ~2 times higher than studies using different sample types for cases and controls (OR 5.12; 95% CI 3.28–8.00). It was found that while the pooled odds ratio was higher there was much more heterogeneity when compared to studies with different samples.

### 3.6 Review of studies not included in the meta‐analyses

There were five studies that were excluded from meta‐analysis. The study by Kingsley et al. (2021) included 80 subjects who were tested for HPV using DNA analysis of exfoliated surface cells. Measure of association could not be estimated because none of the cases or controls tested positive for HPV. Four case–control studies were excluded due to lack of adjusted effect estimates (Chang et al., 2003; Chen et al., 2006; Luo et al., 2007; Szarka et al., 2009). All four studies conducted DNA analysis by PCR. Of these two studies used tissue samples (Chang et al., 2003; Chen et al., 2006) and two used surface cells (Luo et al., 2007; Szarka et al., 2009). The outcomes reported and their effect estimates are presented in Appendix S15. Overall risk of bias was judged to be serious in all five excluded studies.

## 4 DISCUSSION

### 4.1 Summary of results

The results of the meta‐analysis indicate that individuals with oral cancer and OPMDs are more likely to have HPV detected than those who do not have oral cancer. Since most of the studies have a high risk of bias due to confounding and information bias, the pooled estimates should be interpreted with a very high degree of caution as they are likely to lack internal validity.

DNA analysis estimated higher HPV presence than HPV antibody testing. The odds of HPV detection were higher when samples were from exfoliated surface cells and tissues than blood‐based samples. This indicates that strength of association of HPV and OC can vary depending on method of sampling that may lead to bias due to misclassification of the exposure, HPV. DNA analysis showed more consistent results across studies with different sampling methods for cases and controls, but varied widely when the same method was used for both. Thus, despite using a precise molecular detection method, studies employing identical sampling methods for cases and controls may lack precision and consistency. There is a high potential for misclassification due to inappropriate linking of detection method with conclusions of causality. Since none of the studies conducted mRNA analysis, considered the gold standard for measuring oncogenic HPV activity, the true extent of this bias remains unclear, as does the exact role of HPV in OC causality.

### 4.2 Strengths and limitations

A key strength of this systematic review is that it only includes studies where anatomical location of the lesion was clearly specified. Cancers of oropharynx have distinct aetio‐pathological features and HPV has a high affinity for lymphoid tissue, such as tonsils, that are not found in the oral cavity (Gotz et al., 2016; Hübbers & Akgül, 2015). Several studies report that misclassification of oropharyngeal sites like base of tongue and tonsils as OC sites can lead to an overestimation of the association between HPV and OC (Gotz et al., 2016; Hobbs et al., 2006; Hübbers & Akgül, 2015). Treatment methods and prognosis differ based on cancer site. Cancers of oral cavity are typically treated with surgical resection, while oropharyngeal cancers often undergo radiation therapy (Johnson et al., 2020). HPV‐positive cancers generally have better survival rates, but OPCs tend to metastasize more to lymph nodes, resulting in lower survival rates compared to OC (Moro et al., 2018; Sathish et al., 2014). Focusing solely on oral cavity cancers in this study has helped mitigate bias from outcome misclassification.

Another strength is that only studies reporting measures of association, such as odds ratio, were included. By comparing the outcome of interest in two or more exposure groups, measures of association can quantify the strength of the overall effect of HPV on oral cancer. They allow for examination of the causal effects based on assessment of confounding, selection, and information bias, while studies that do not quantify associations do not. Examination of the causal effect of HPV on OC is important for interventions such as vaccination.

Additionally, the study designs and effect estimate measures were homogenous and we were successfully able to conduct a meta‐analysis. We were also able to examine the source of heterogeneity in effect estimates by undertaking a sub‐group analysis according to the method by which HPV was measured in cases and controls. This review also used the ROBINS‐E tool, which is the preferred quality assessment tool for risk of bias assessment.

The meta‐analysis could not be restricted to low risk of bias studies as all included studies were case control studies and had moderate‐to‐severe risk of bias. Although the ROBINS‐E is the preferred tool for risk of bias in observational studies, it has been designed for cohort studies. It is not tailored for use in case–control studies, and this may have affected judgements made. The gold standard for indicating clinically relevant HPV presence is the evidence of transcriptional activation of viral oncoproteins which can be determined by mRNA analysis (Bishop et al., 2012; Jung et al., 2010). Research on HPV‐driven OPCs indicates that mRNA analysis, as opposed to other methods like p16 expression or DNA expression via ISH or PCR, is more effective in detecting HPV‐driven OPCs, especially those driven by HPV 16 (Holzinger et al., 2012; Randén‐Brady et al., 2019). We acknowledge that none of the included studies used gold standard mRNA testing. While it is not a limitation of this review, it is a gap in existing literature and the absence of these studies could lead to lack of clarity on the effect of HPV on oral cancer.

### 4.3 Similarities and dissimilarities with literature

Current finding that individuals with OC are more likely to have HPV detected than healthy controls was in accordance with various existing reviews (Chaitanya et al., 2016; Hobbs et al., 2006; Syrjanen et al., 2011). However, the strength of association in the present review is stronger than previously reported. Some differences may be attributed to inclusion of other head and neck sites besides the oral cavity (Chaitanya et al., 2016) or lack of information about anatomical sites (Syrjanen et al., 2011), limiting exposure to HPV 16 only (Hobbs et al., 2006) or use of crude estimates instead of adjusted estimates examined in present study (Chaitanya et al., 2016; Hobbs et al., 2006; Syrjanen et al., 2011). It was also found that individuals with OPMDs were more likely to have HPV detection than healthy controls. This was consistent with the results reported by existing studies (Ma et al., 2016; Shang et al., 2020; Syrjanen et al., 2011).

Studies with DNA analysis had higher odds of HPV detection than studies reporting HPV antibodies, possibly due to differences in sensitivity and specificity between the two techniques (Hobbs et al., 2006). Since DNA detection broadly suggests viral presence, while serological E6 antibodies suggest stronger support for pathological HPV activity, a larger number of DNA‐positive samples should be proportionally observed. Tissue tropism, which refers to the ability of a virus to infect distinct tissue types and ultimately establish a successful infection may also play a crucial role (McFadden et al., 2009). Research suggests that while serological testing of HPV16 E6 antibody is a highly sensitive diagnostic marker for HPV16 caused OPSCC, it might be less sensitive for HPV16 related cancers for sites with less HPV tropism than the oropharynx (Holzinger et al., 2017). It was found that cell and tissue‐based samples had higher odds of HPV positivity than blood‐based samples, which is likely for the same reasons as just suggested. Interestingly, this finding was in contrast to that reported by Chaitanya et al. (2016) where serological samples detected HPV exposure 1.129 times greater than tissue‐based DNA testing. There was high inconsistency in the effect estimates of studies that conducted DNA analysis using the same method for cases and controls. This might be attributed to small sample sizes or low ratio of controls to cases.

### 4.4 Research and policy implications

The absence of gold standard mRNA testing in the selected studies signifies a notable research gap. It highlights an opportunity for future research to incorporate this advanced testing method. Closing this gap could enhance our understanding of the precise effects of HPV on oral cancer. The current review highlights the need for more research on the validity of methods measuring and pre‐concluding causal HPV detection in oral cavity sites when tested against gold standard mRNA testing. Risk of bias analysis findings indicate that current estimates are not reliable due to high confounding. Therefore, well‐designed cohort studies which account for key confounding factors are needed to generate estimates of causal effects of HPV on OC. To avoid potential overestimation of the role of HPV in OC, studies must avoid clubbing oral and oropharyngeal cancer together when looking at HPV effect. Further, more research from low‐ and middle‐income countries is needed to increase external validity of findings since current evidence is primarily from high‐income countries.

Current findings may impact policies relating to anticipated OC prevention through vaccination as the role of HPV in OC needs to be reconsidered in light of the current limited knowledge on the causal effect of HPV. Estimating causal effect is critical to quantify population‐level contribution of HPV on OC and compare it with other well‐established risk factors: alcohol use, chewing tobacco, and cigarette smoking. Otherwise, finite resources currently allocated for OC prevention may be misallocated.

## 5 CONCLUSION

When site of oral cancer is clearly differentiated between oropharynx and oral cavity, there is a strong association in the current literature between HPV detection and OC with 12 studies showing higher odds of HPV in OC and our meta‐analysis indicating that cases with OC are 5 times more likely to test positive for HPV compared to controls. However, there is a lack of studies that account for confounding, gold standard testing methods to conclude causality and consistent measurements for cases and control groups that are important for reducing bias. Given the uncertainty surrounding the causal involvement of HPV in oral cancer, it is imperative from a policy standpoint to ascertain the precise impact of HPV on OC in comparison to well‐established risk factors like tobacco and alcohol use. This is essential for guiding effective public health interventions such as HPV vaccination programs and ensuring the appropriate allocation of resources, particularly for prevention of oral cancers, given the rise in incidence of tongue cancers seen in countries (Satgunaseelan et al., 2020). This systematic review and meta‐analysis highlights that there is currently insufficient high‐quality evidence of the causal effect of HPV on cancers of the oral cavity underscoring the need for methodological rigour and consistency in future research.

## AUTHOR CONTRIBUTIONS

**Gagandeep Kaur:** Conceptualization; methodology; formal analysis; visualization; project administration; writing – original draft; data curation. **Tami Yap:** Conceptualization; methodology; supervision; writing – review and editing. **Rishi Ramani:** Methodology; writing – review and editing; data curation. **Michael McCullough:** Writing – review and editing. **Ankur Singh:** Conceptualization; methodology; project administration; writing – review and editing; supervision; formal analysis.

## CONFLICT OF INTEREST STATEMENT

All authors have no conflicts of interest to disclose.

## PATIENT AND PUBLIC INVOLVEMENT

Patients and the public were not involved in the design, or conduct, or reporting, or dissemination plans of the research.

## Supporting information


Data S1.

## Data Availability

The data that supports the findings of this study are available in the supplementary material of this article.
