# Supplementary material for: Assessing bias in the causal role of HPV in oral cancer: A systematic review and meta‐analysis
Source: Oral Dis. 2024 Jul 2;30(8):5379–87. doi: 10.1111/odi.15062 (PMC11610703; doi:10.1111/odi.15062)
Supplement: Supplementary file 1 — Data S1. [file ODI-30-5379-s001.docx]

**Supplementary Appendix**

**Appendix 1** Summary of systematic reviews on HPV and oral cancer

| **Author year** | **Aim** | **Results** | **Conclusion** |
| --- | --- | --- | --- |
| Hobbs et al. (2006) | HPV risk variation according to anatomical site within the **upper**  **aero-digestive tract.** | The association between HPV16 and cancer was strongest for tonsil (OR: 15.1, 95% CI: 6.8–33.7), intermediate for oropharynx (OR: 4.3, 95% CI: 2.1–8.9) and **weakest for oral** (OR: 2.0, 95% CI: 1.2–3.4) and larynx (OR: 2.0, 95% CI: 1.0–4.2). | Strongest and most consistent association is with tonsil cancer,  However, the method of viral detection may be an important source of heterogeneity |
| Syrjanen et al. (2011) | To calculate pooled risk estimates for the association of HPV with **OSCC and OPMD.** To examine the effects of sampling techniques on HPV detection rates. | Significant association was found between pooled HPV-DNA detection and OSCC (OR = 3.98; 95% CI: 2.62-6.02) and even for HPV16 only (OR = 3.86; 95% CI: 2.16-6.86).  HPV was also associated with OPMD (OR = 3.87; 95% CI: 2.87-5.21). | The results suggest a potentially **important causal association** between HPV and OSCC and OPMD. |
| Kansy et al. (2014) | To evaluate the role of high-risk HPV types in initiation and progression of **OSCC.** | Overall HPV prevalence of 30.1 % in OSCCs. The most frequently identified subtypes were HPV-16 and HPV-18 (25.4 and 18.1 %, respectively).  In general, no definite role of high-risk HPV is currently deducible from the literature. | Role of HPV as a causative agent is less clear than the role in oropharyngeal tumours. The infection **might not be the cause of carcinogenesis** in a significant number of patients. |
| Chaitanya et al. (2016) | Evaluating the presence of HPV and **oral and oropharyngeal cancers** | OR was 2.82, indicating increased risk of HPV among cases when compared to controls. | Potentially significant **casual relation** between HPV and oral and oropharyngeal cancers. |
| Smitha et al. (2017) | To address the association of p16^INK4a^ and HPV DNA with **OSCC.** | 559/3339 OSCC patients were diagnosed with the presence of HPV16 DNA with a random proportion of 20.1% at 95% confidence interval (CI) (13.9-27.1). Overexpression of p16^INK4a^ was observed in 709 patients with a random proportion of 25.4% at 95% CI (14.3-38.3). | The frequency of HPV infection is **significantly higher** in patients with OSCC. |
| Villa et al. (2020) | To summarize the evidence on safety, efficacy, and effectiveness of **HPV vaccines** in the general population. | Compared with placebo or non-HPV-type vaccine group, HPV-vaccinated participants had statistically significantly lower rates of HPV infection and condylomata lesions, and decreased rates of HPV-related precancerous lesions, which did not always attain statistical significance | HPV vaccines are safe, effective, and **efficacious against** vaccine-type HPV infection and HPV-associated cellular changes, including **precancerous and benign lesions**. |
| Melo et al. (2021) | To evaluate the relationship between HPV infection and **OSCC** | 17 out of 383 (4.4%) OSCC patients were HPV/mRNA-positive, | Association of HPV and OSCC was **not assessed** as cross-sectional studies lacked control group (no longitudinal studies). |

**Appendix 2** Different types of sampling and detection methods of measuring HPV

**Appendix 3: PRISMA checklist**

| **Section and Topic** | **Item #** | **Checklist item** | **Location where item is reported** |
| --- | --- | --- | --- |
| **TITLE** | | |  |
| Title | 1 | Identify the report as a systematic review. | Page 1 |
| **ABSTRACT** | | |  |
| Abstract | 2 | See the PRISMA 2020 for Abstracts checklist. | Page 3 |
| **INTRODUCTION** | | |  |
| Rationale | 3 | Describe the rationale for the review in the context of existing knowledge. | Page 6-7 |
| Objectives | 4 | Provide an explicit statement of the objective(s) or question(s) the review addresses. | Page 7 |
| **METHODS** | | |  |
| Eligibility criteria | 5 | Specify the inclusion and exclusion criteria for the review and how studies were grouped for the syntheses. | Page 7 |
| Information sources | 6 | Specify all databases, registers, websites, organisations, reference lists and other sources searched or consulted to identify studies. Specify the date when each source was last searched or consulted. | Page 8 |
| Search strategy | 7 | Present the full search strategies for all databases, registers and websites, including any filters and limits used. | Page 8 |
| Selection process | 8 | Specify the methods used to decide whether a study met the inclusion criteria of the review, including how many reviewers screened each record and each report retrieved, whether they worked independently, and if applicable, details of automation tools used in the process. | Page 8 |
| Data collection process | 9 | Specify the methods used to collect data from reports, including how many reviewers collected data from each report, whether they worked independently, any processes for obtaining or confirming data from study investigators, and if applicable, details of automation tools used in the process. | Page 8 |
| Data items | 10a | List and define all outcomes for which data were sought. Specify whether all results that were compatible with each outcome domain in each study were sought (e.g. for all measures, time points, analyses), and if not, the methods used to decide which results to collect. | Page 8 |
|  | 10b | List and define all other variables for which data were sought (e.g. participant and intervention characteristics, funding sources). Describe any assumptions made about any missing or unclear information. | Page 8 |
| Study risk of bias assessment | 11 | Specify the methods used to assess risk of bias in the included studies, including details of the tool(s) used, how many reviewers assessed each study and whether they worked independently, and if applicable, details of automation tools used in the process. | Page 8 |
| Effect measures | 12 | Specify for each outcome the effect measure(s) (e.g. risk ratio, mean difference) used in the synthesis or presentation of results. | Page 8 |
| Synthesis methods | 13a | Describe the processes used to decide which studies were eligible for each synthesis (e.g. tabulating the study intervention characteristics and comparing against the planned groups for each synthesis (item #5)). | Page 8 |
|  | 13b | Describe any methods required to prepare the data for presentation or synthesis, such as handling of missing summary statistics, or data conversions. | Page 9 |
|  | 13c | Describe any methods used to tabulate or visually display results of individual studies and syntheses. | Page 9 |
|  | 13d | Describe any methods used to synthesize results and provide a rationale for the choice(s). If meta-analysis was performed, describe the model(s), method(s) to identify the presence and extent of statistical heterogeneity, and software package(s) used. | Page 9 |
|  | 13e | Describe any methods used to explore possible causes of heterogeneity among study results (e.g. subgroup analysis, meta-regression). | Page 9 |
|  | 13f | Describe any sensitivity analyses conducted to assess robustness of the synthesized results. |  |
| Reporting bias assessment | 14 | Describe any methods used to assess risk of bias due to missing results in a synthesis (arising from reporting biases). | Page 8 |
| Certainty assessment | 15 | Describe any methods used to assess certainty (or confidence) in the body of evidence for an outcome. | Page 8 |
| **RESULTS** | | |  |
| Study selection | 16a | Describe the results of the search and selection process, from the number of records identified in the search to the number of studies included in the review, ideally using a flow diagram. | Page 9 |
|  | 16b | Cite studies that might appear to meet the inclusion criteria, but which were excluded, and explain why they were excluded. | Page 9 |
| Study characteristics | 17 | Cite each included study and present its characteristics. | Page 9-10 |
| Risk of bias in studies | 18 | Present assessments of risk of bias for each included study. | Page 9-10 |
| Results of individual studies | 19 | For all outcomes, present, for each study: (a) summary statistics for each group (where appropriate) and (b) an effect estimate and its precision (e.g. confidence/credible interval), ideally using structured tables or plots. | Page 10 |
| Results of syntheses | 20a | For each synthesis, briefly summarise the characteristics and risk of bias among contributing studies. | Page 10 |
|  | 20b | Present results of all statistical syntheses conducted. If meta-analysis was done, present for each the summary estimate and its precision (e.g. confidence/credible interval) and measures of statistical heterogeneity. If comparing groups, describe the direction of the effect. | Page 10-11 |
|  | 20c | Present results of all investigations of possible causes of heterogeneity among study results. | Page 10-11 |
|  | 20d | Present results of all sensitivity analyses conducted to assess the robustness of the synthesized results. | - |
| Reporting biases | 21 | Present assessments of risk of bias due to missing results (arising from reporting biases) for each synthesis assessed. | - |
| Certainty of evidence | 22 | Present assessments of certainty (or confidence) in the body of evidence for each outcome assessed. | Page 10 |
| **DISCUSSION** | | |  |
| Discussion | 23a | Provide a general interpretation of the results in the context of other evidence. | Page 11 |
|  | 23b | Discuss any limitations of the evidence included in the review. | Page 12 |
|  | 23c | Discuss any limitations of the review processes used. | Page 12-13 |
|  | 23d | Discuss implications of the results for practice, policy, and future research. | Page 12-13 |
| **OTHER INFORMATION** | | |  |
| Registration and protocol | 24a | Provide registration information for the review, including register name and registration number, or state that the review was not registered. | Page 7 |
|  | 24b | Indicate where the review protocol can be accessed, or state that a protocol was not prepared. | Page 7 |
|  | 24c | Describe and explain any amendments to information provided at registration or in the protocol. | - |
| Support | 25 | Describe sources of financial or non-financial support for the review, and the role of the funders or sponsors in the review. | Page 1 |
| Competing interests | 26 | Declare any competing interests of review authors. | Page 2 |
| Availability of data, code and other materials | 27 | Report which of the following are publicly available and where they can be found: template data collection forms; data extracted from included studies; data used for all analyses; analytic code; any other materials used in the review. | Appendix |

*From:*  Page MJ, McKenzie JE, Bossuyt PM, Boutron I, Hoffmann TC, Mulrow CD, et al. The PRISMA 2020 statement: an updated guideline for reporting systematic reviews. BMJ 2021;372:n71. doi: 10.1136/bmj.n71

For more information, visit: <http://www.prisma-statement.org/>

**Appendix 4** Search terms for PubMed

| **Exposure** | HPV [TIAB] or human papilloma*[TIAB] |
| --- | --- |
| **Site** | mouth [MH] or tongue or palate or lingual or buccal or retromolar or cheek or gingiva or intra-oral |
| **Outcome** | neoplasms [MH] or precancerous conditions [MH] or hyperplas* or ulcer or nodul* or metasta* or precancer* or dysplasia or premalignant or pre-malignant or oral squamous cell carcinoma[TIAB] or oral potentially malignant disorder* [TIAB] or oral potentially malignant lesion*[TIAB] or leukoplakia or white patches or erythroplakia or red patches or erythroleukoplakia or lichen planus or oral or submucous fibrosis or oral lupus erythromatosus or oral lichenoid |
| **Site + outcome** | mouth neoplasms [MH] |

**Appendix 5** Search terms for MEDLINE, PsycInfo and Embase

| **Exposure** | HPV.ti,ab. or human papilloma*.ti,ab. |
| --- | --- |
| **Site** | exp Mouth/ or tongue.mp.or palate.mp.or lingual.mp.or buccal.mp.or retromolar.mp. or cheek.mp. or gingiva.mp. or intra-oral.mp. |
| **Outcome** | exp neoplasms/ or exp precancerous conditions/ or hyperplas*.mp. or ulcer.mp. or nodul*.mp. or metasta*.mp. or precancer*.mp. or dysplasia.mp. or premalignant.mp. or pre-malignant.mp. or “oral squamous cell carcinoma”.ti,ab or “oral potentially malignant disorder*”.ti,ab or “oral potentially malignant lesion*”.ti,ab or leukoplakia.mp. or white patches.mp. or erythroplakia.mp. or red patches.mp. or erythroleukoplakia.mp. or lichen planus.mp. or oral submucous fibrosis.mp. or oral lupus erythromatosus.mp. or oral lichenoid.mp |
| **Site + outcome** | exp mouth neoplasms/ |

**Appendix 6** Data Collection Form

**­­**

**General Information**

| Date completed |  |
| --- | --- |
| Reviewer |  |

**Identification**

| Title |  |
| --- | --- |
| Paper ID |  |
| Notes |  |

**Characteristics of studies**

| Aim of study according to authors |  | | | | |
| --- | --- | --- | --- | --- | --- |
| Study design according to authors |  | | | | |
| Place (country) |  | | | | |
| Ethics approval | Yes | | No | | Unclear |
| Informed consent | Yes | | No | | Unclear |
| Exposure |  | | | | |
| Outcome |  | | | | |
|  | **Cases** | | | **Controls** | |
| Total number participants |  | | |  | |
| Age range |  | | |  | |
| Sex |  | | |  | |
| Matching of controls |  | | |  | |
| Inclusion criteria |  | | |  | |
| Exclusion criteria |  | | |  | |
| Site of lesion |  | | | - | |
| Diagnostic method |  | | |  | |
| Sampling method |  | | |  | |
|  |  | | |  | |
| Covariates |  | | | | |
| Results | crude |  | | | |
|  | adjusted |  | | | |
| Limitations according to author |  | | | | |
| Conclusions |  | | | | |
| Notes |  | | | | |


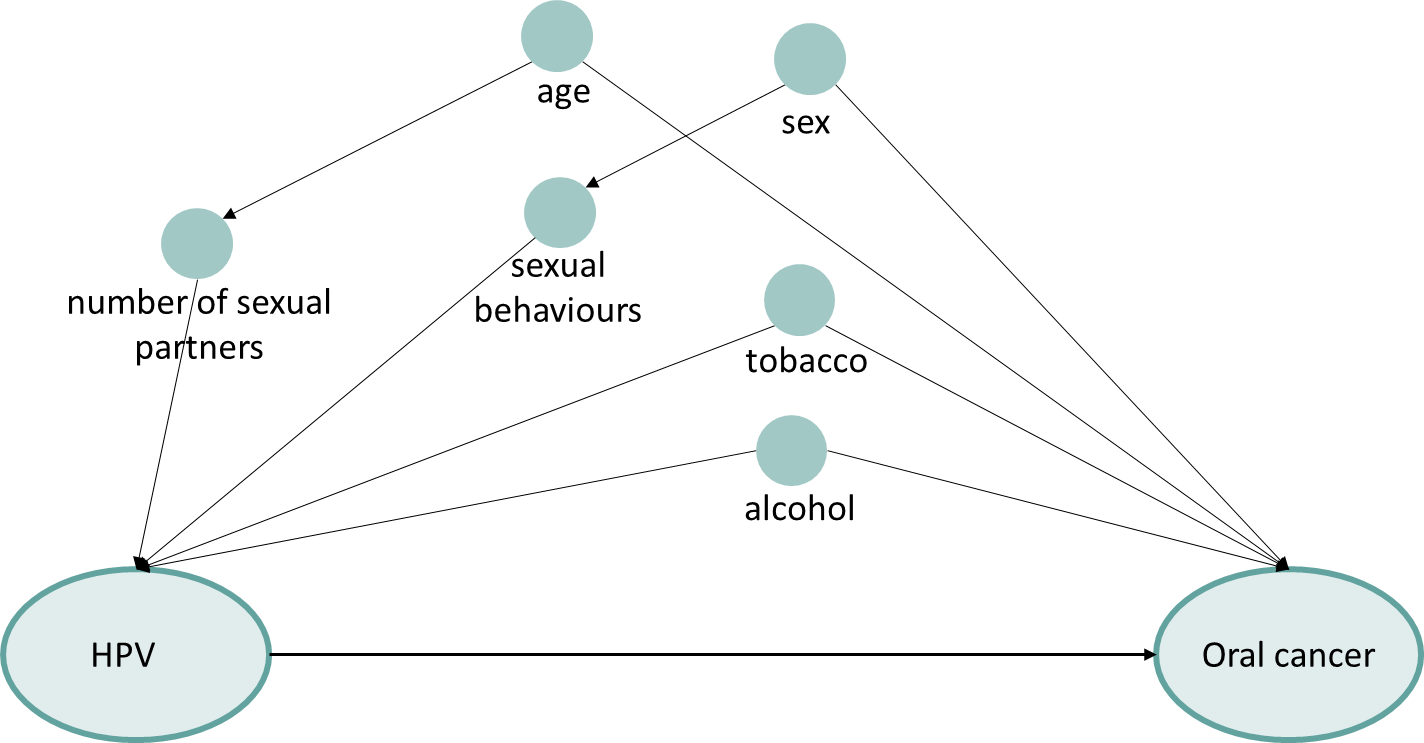


**Appendix 7** Directed acyclic graph showing confounder selection and a causal association between HPV and oral cancer.


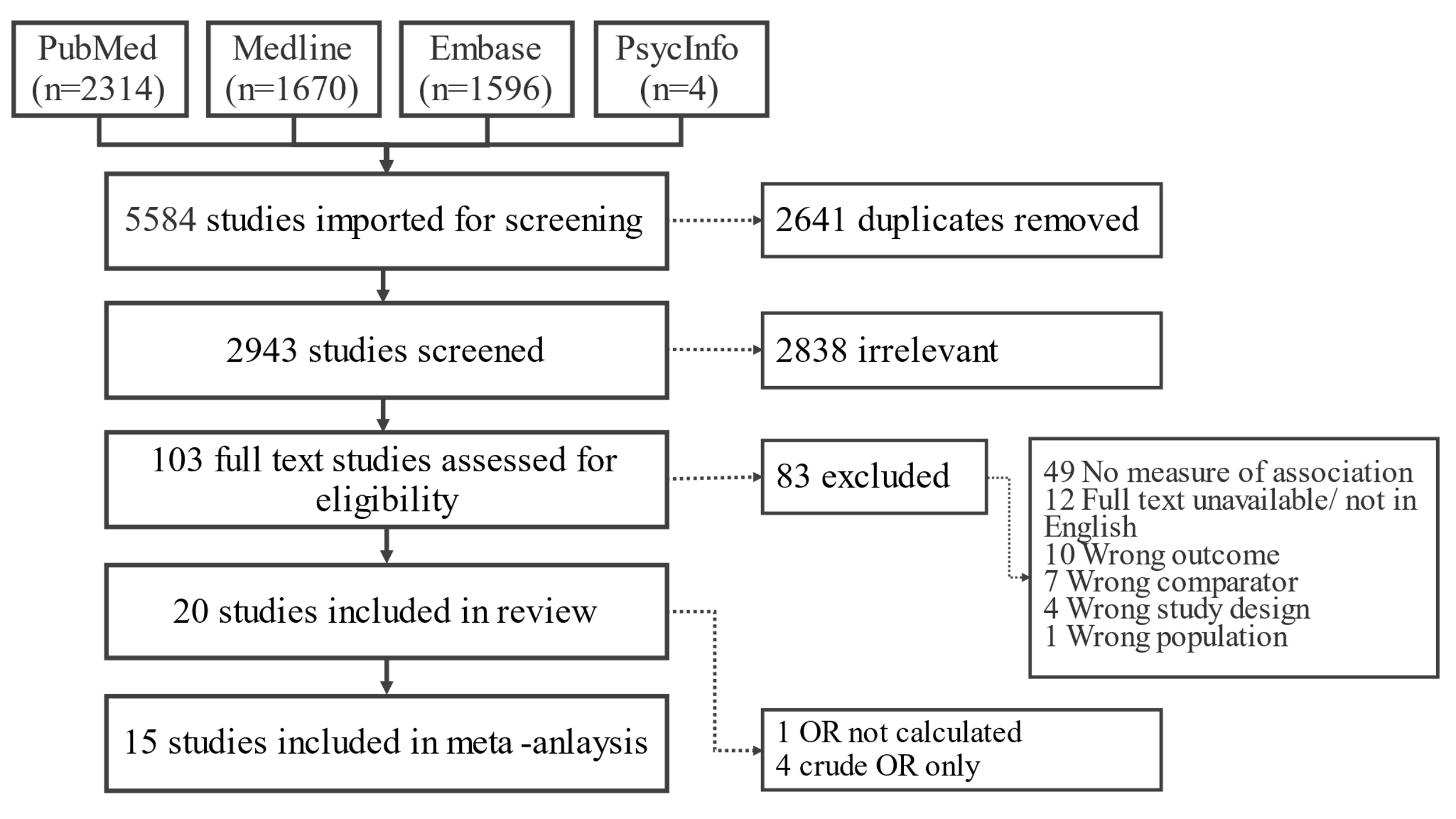


**Appendix 8:** Prisma flow chart of study selection

**Appendix 9** Risk of bias assessment using ROBINS-E

| **First author (publication year)** | **Bias due to confounding** | **Bias in selection of participants into the study** | **Bias in classification of exposures** | **Bias due to departures from intended exposures** | **Bias due to missing data** | **Bias in measurement of outcomes** | **Bias in selection of the reported result** | **Study-level RoB judgement** |
| --- | --- | --- | --- | --- | --- | --- | --- | --- |
| Anaya-Saavedra 2008 | Serious | Moderate | Moderate | Low | Low | Low | Low | **Serious** |
| Applebaum 2007 | Serious | Moderate | Serious | Low | Low | Low | Low | **Serious** |
| Chen 2002 | Serious | Serious | Moderate | Low | Low | Moderate | Moderate | **Serious** |
| Chen 2016 | Moderate | Moderate | Moderate | Low | Low | Low | Moderate | **Moderate** |
| Giovanneli 2002 | Moderate | Serious | Moderate | Low | Low | Moderate | Low | **Serious** |
| Gonzalez-Ramírez 2013 | Serious | Moderate | Moderate | Low | Low | Low | Low | **Serious** |
| Hansson 2005 | Serious | Moderate | Serious | Low | Low | Low | Moderate | **Serious** |
| Kerishnan 2016 | Serious | Serious | Serious | Low | Low | Low | Low | **Serious** |
| Majumder 2009 | Moderate | Serious | Moderate | Low | Low | Moderate | Moderate | **Serious** |
| Mork 2001 | Serious | Moderate | Serious | Low | Low | Low | Serious | **Serious** |
| Pintos 2008 | Moderate | Moderate | Moderate | Low | Low | Low | Moderate | **Moderate** |
| Ribeiro 2011 | Moderate | Moderate | Serious | Low | Low | Low | Moderate | **Serious** |
| Saini 2011 | Moderate | Serious | Moderate | Low | Low | Low | Low | **Serious** |
| Tsimplaki 2017 | Moderate | Serious | Moderate | Low | Low | Low | Low | **Serious** |
| DallaTorre 2015 | Serious | Serious | Moderate | Low | Low | Low | Moderate | **Serious** |
| Chang 2003 | Serious | Serious | Moderate | Low | Low | Low | Low | **Serious** |
| Chen 2006 | Serious | Serious | Moderate | Low | Low | Moderate | Moderate | **Serious** |
| Luo 2007 | Serious | Serious | Serious | Low | Low | Low | Low | **Serious** |
| Szarka 2009 | Serious | Moderate | Serious | Low | Low | Low | Low | **Serious** |
| Kingsley 2021 | Serious | Moderate | Moderate | Low | Low | Low | Low | **Serious** |


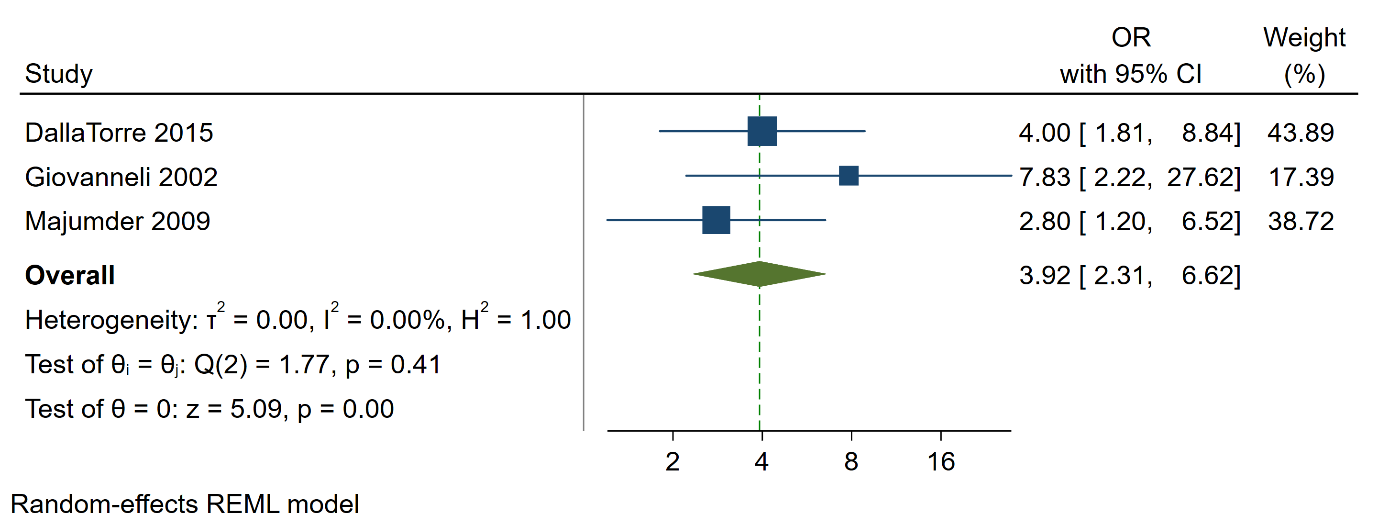


**Appendix 10** Forrest plot showing the pooled effect of HPV on OPMDs. Data are presented as odds ratio for each study (blue boxes), 95% CIs (horizontal lines) and summary as odds ratio with 95% CI (green diamond).


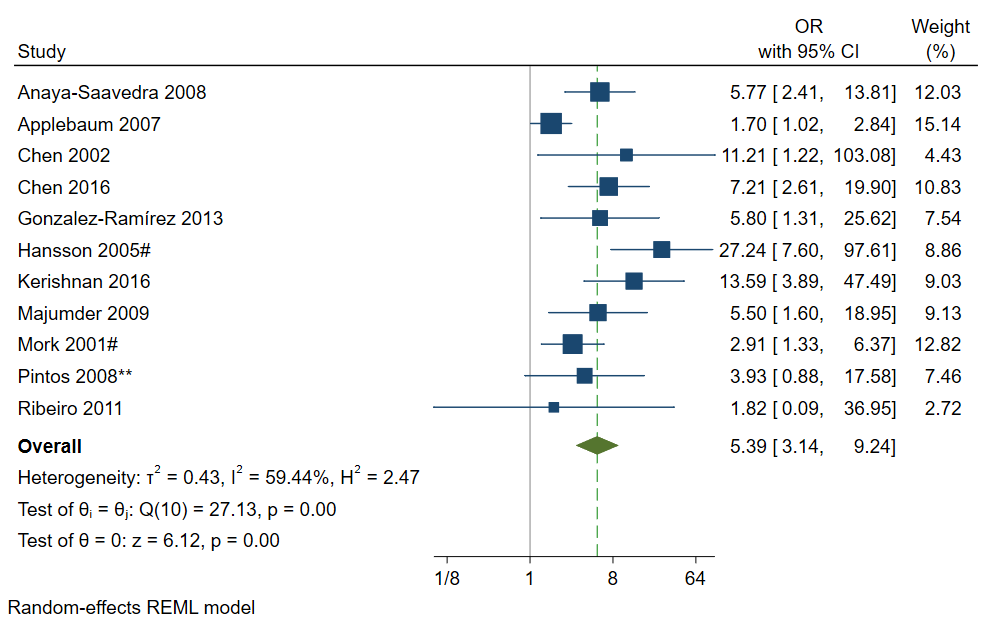


**Appendix 11** Forrest plot showing the pooled effect of high risk HPV only/HPV16/HPV18 on oral cancer. Data are presented as odds ratio for each study (blue boxes), 95% CIs (horizontal lines) and summary as odds ratio with 95% CI (green diamond).

**Blood based sample; # Pooled oral tongue, FOM, other oral cavity


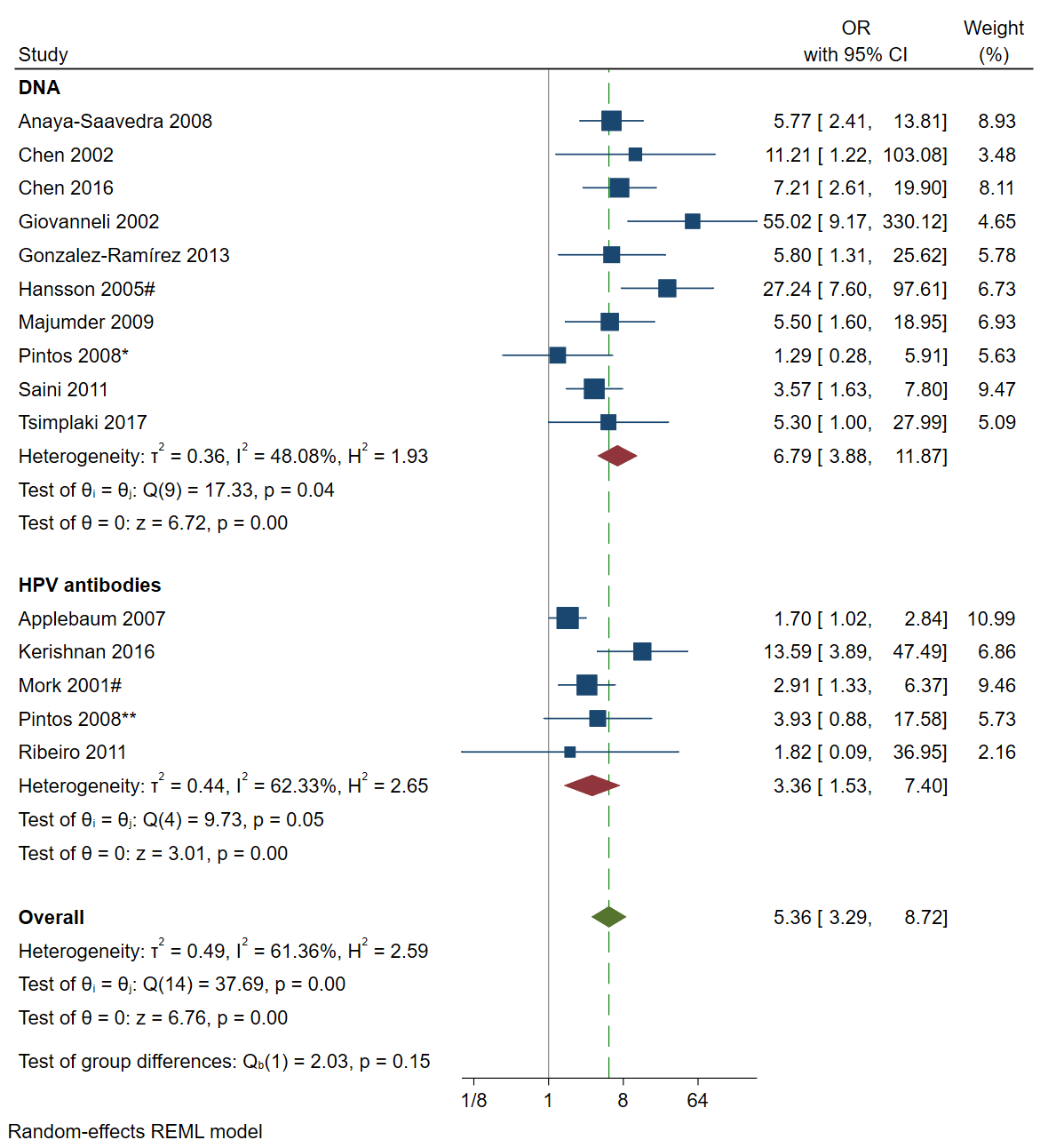


**Appendix 12** Forrest plot showing the pooled effect of HPV on oral cancer, grouped by detection method. Data are presented as odds ratio for each study (blue boxes), 95% CIs (horizontal lines), subgroup pooled odds ratio with 95% CI (red diamonds) and overall summary as odds ratio with 95% CI (green diamond).


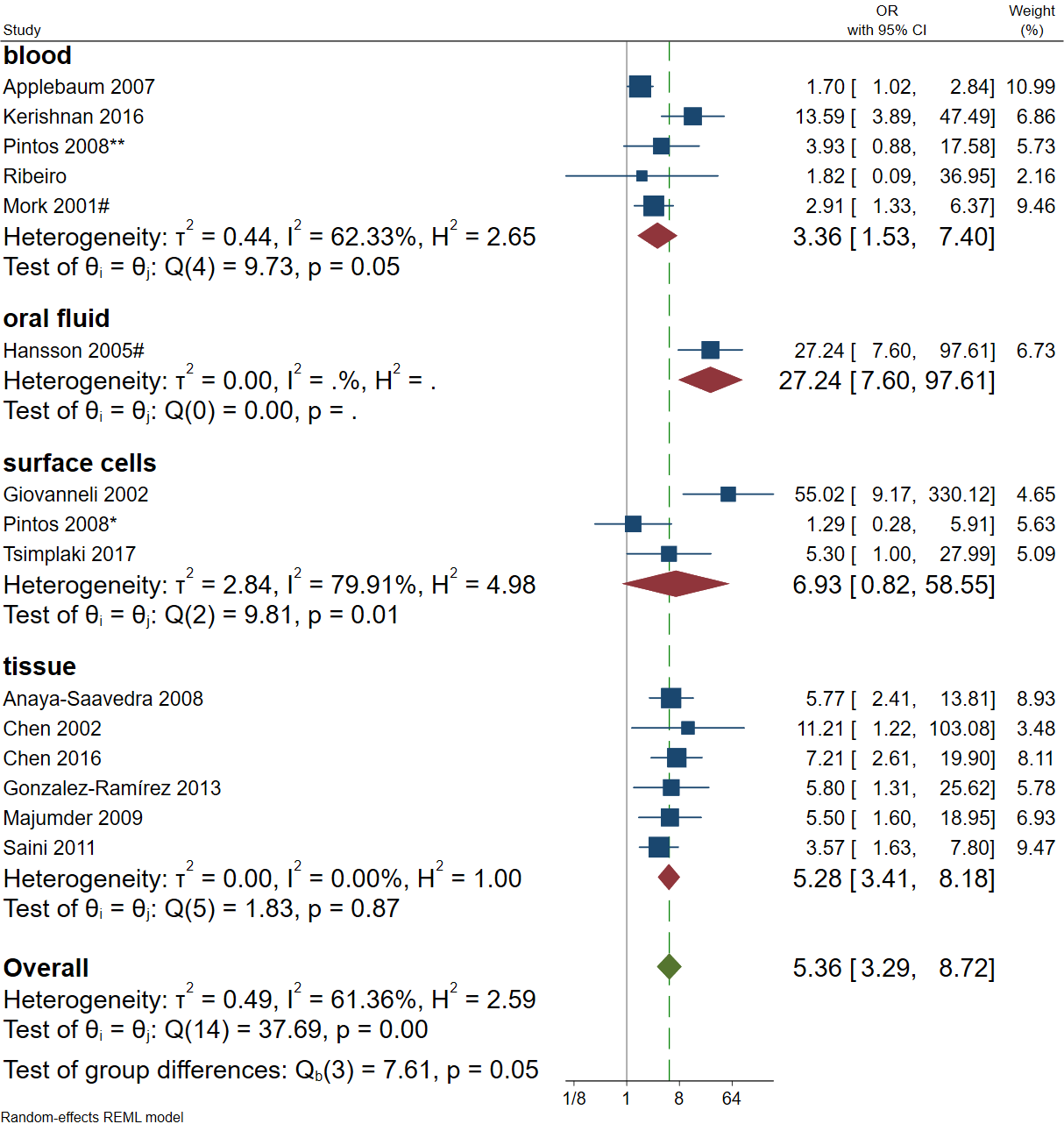


**Appendix 13** Forrest plot showing the pooled effect of HPV on oral cancer, grouped by sampling method for cases. Data are presented as odds ratio for each study (blue boxes), 95% CIs (horizontal lines), subgroup pooled odds ratio with 95% CI (red diamonds) and overall summary as odds ratio with 95% CI (green diamond).


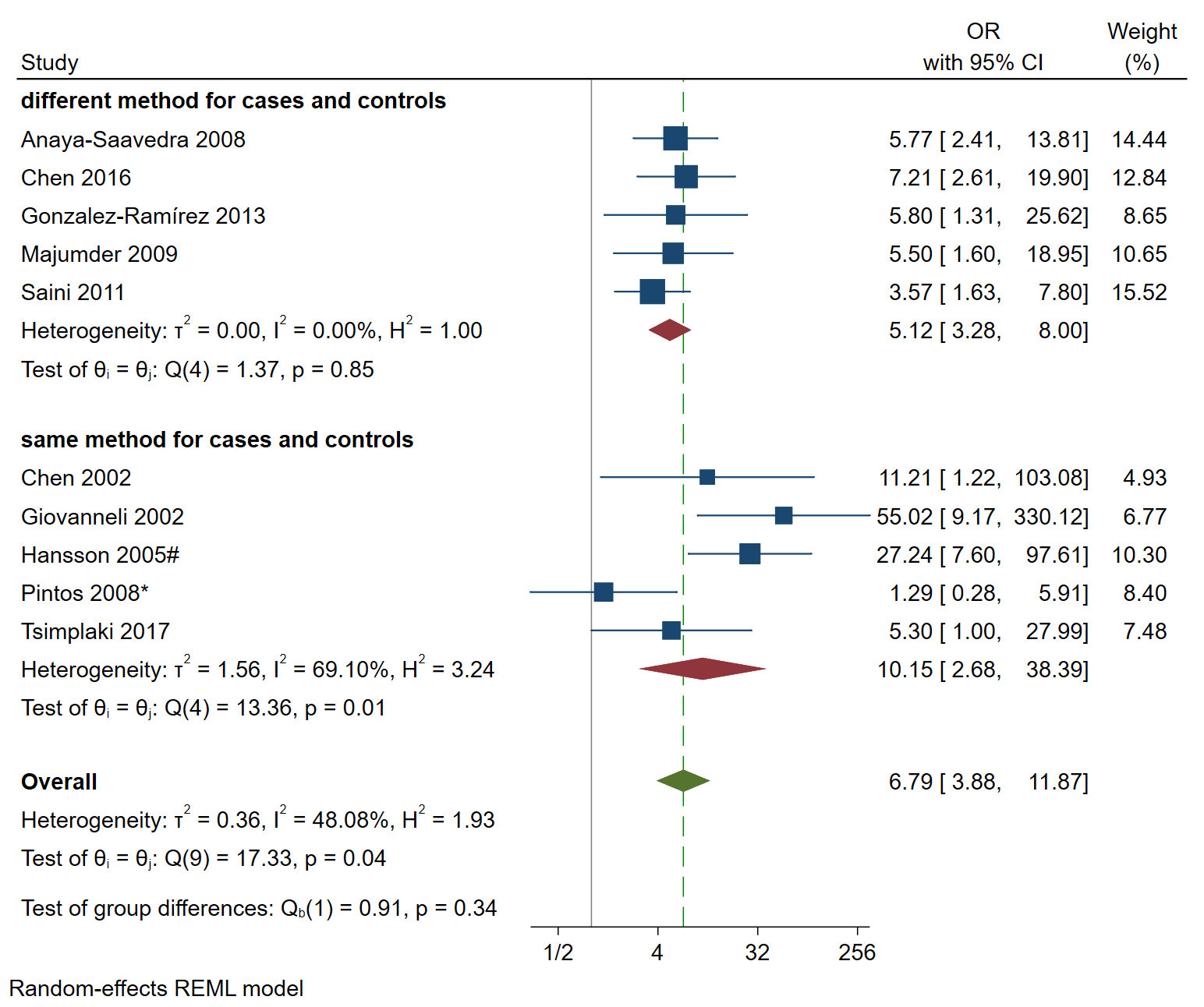


**Appendix 14** Forrest plot showing the pooled effect of HPV on oral cancer, for studies that reported DNA analysis as detection method, grouped by use of same versus different sampling method for cases and controls. Data are presented as odds ratio for each study (blue boxes), 95% CIs (horizontal lines), subgroup pooled odds ratio with 95% CI (red diamonds) and overall summary as odds ratio with 95% CI (green diamond).

**Appendix 15** Study characteristics of studies excluded from meta-analysis

| Author date | Exposure | Outcome | Odds ratio(95% CI) |
| --- | --- | --- | --- |
| Chang et al. (2003) | HPV | OSCC | 3.92 (1.48-10.39) |
| Chen et al. (2006) | HPV 16 | OPMDs:  Submucosal fibrosis  Leukoplakia  Squamous papilloma | 2.06 (0.45-9.3)  2.74 (0.81-9.31)  1.9 (0.52-6.96) |
| Luo et al. (2007) | HPV | OSCC  OPMDs | 2.22 (0.93-5.34)  2.84 (1.19-6.82) |
| Szarka et al. (2009) | HPV | OSCC  OPMDs | 20.97(5.98-73.05)  15.92 (4.33-59.59) |

**References**

Chaitanya, N. C., Allam, N. S., Gandhi Babu, D. B., Waghray, S., Badam, R. K., & Lavanya, R. (2016). Systematic meta-analysis on association of human papilloma virus and oral cancer. *J Cancer Res Ther, 12*(2), 969-974. doi:10.4103/0973-1482.179098

Chang, J. Y., Lin, M. C., & Chiang, C. P. (2003). High-risk human papillomaviruses may have an important role in non-oral habits-associated oral squamous cell carcinomas in Taiwan. *Am J Clin Pathol, 120*(6), 909-916. doi:10.1309/C5P6-NUQ2-NW6L-CTBP

Chen, P. C., Pan, C. C., Kuo, C., & Lin, C. P. (2006). Risk of oral nonmalignant lesions associated with human papillomavirus infection, betel quid chewing, and cigarette smoking in Taiwan: an integrated molecular and epidemiologic study. *Arch Pathol Lab Med, 130*(1), 57-61. doi:10.5858/2006-130-57-ROONLA

Hobbs, C. G., Sterne, J. A., Bailey, M., Heyderman, R. S., Birchall, M. A., & Thomas, S. J. (2006). Human papillomavirus and head and neck cancer: a systematic review and meta-analysis. *Clin Otolaryngol, 31*(4), 259-266. doi:10.1111/j.1749-4486.2006.01246.x

Kansy, K., Thiele, O., & Freier, K. (2014). The role of human papillomavirus in oral squamous cell carcinoma: myth and reality. *Oral Maxillofac Surg, 18*(2), 165-172. doi:10.1007/s10006-012-0383-0

Luo, C. W., Roan, C. H., & Liu, C. J. (2007). Human papillomaviruses in oral squamous cell carcinoma and pre-cancerous lesions detected by PCR-based gene-chip array. *Int J Oral Maxillofac Surg, 36*(2), 153-158. doi:10.1016/j.ijom.2006.09.005

Melo, B. A. C., Vilar, L. G., Oliveira, N. R., Lima, P. O., Pinheiro, M. B., Domingueti, C. P., & Pereira, M. C. (2021). Human papillomavirus infection and oral squamous cell carcinoma - a systematic review. *Braz J Otorhinolaryngol, 87*(3), 346-352. doi:10.1016/j.bjorl.2020.10.017

Smitha, T., Mohan, C. V., & Hemavathy, S. (2017). Prevalence of human papillomavirus16 DNA and p16(INK4a) protein in oral squamous cell carcinoma: A systematic review and meta-analysis. *Journal of oral and maxillofacial pathology : JOMFP, 21*(1), 76-81. doi:10.4103/jomfp.JOMFP_248_16

Syrjanen, S., Lodi, G., von Bultzingslowen, I., Aliko, A., Arduino, P., Campisi, G., . . . Jontell, M. (2011). Human papillomaviruses in oral carcinoma and oral potentially malignant disorders: a systematic review. *Oral Dis, 17 Suppl 1*, 58-72. doi:10.1111/j.1601-0825.2011.01792.x

Szarka, K., Tar, I., Fehér, E., Gáll, T., Kis, A., Tóth, E. D., . . . Gergely, L. (2009). Progressive increase of human papillomavirus carriage rates in potentially malignant and malignant oral disorders with increasing malignant potential. *Oral Microbiol Immunol, 24*(4), 314-318. doi:10.1111/j.1399-302X.2009.00516.x

Villa, A., Patton, L. L., Giuliano, A. R., Estrich, C. G., Pahlke, S. C., O'Brien, K. K., . . . Araujo, M. W. B. (2020). Summary of the evidence on the safety, efficacy, and effectiveness of human papillomavirus vaccines: Umbrella review of systematic reviews. *J Am Dent Assoc, 151*(4), 245-254 e224. doi:10.1016/j.adaj.2019.10.010
